# Optical coherence tomography biomarkers of posterior scleritis in different stages of the disease activity

**DOI:** 10.3389/fopht.2026.1789004

**Published:** 2026-07-10

**Authors:** Sameera Nayak, Niroj Kumar Sahoo, Udit Ajmani, P. Srinivas Rao, Raja Narayanan, Ritesh Narula, Rajeev R. Pappuru

**Affiliations:** 1 Anant Bajaj Retina Institute, L V Prasad Eye Institute, Vijayawada/Hyderabad/Bhubaneswar/Vishakhapatnum/Kadapa, India; 2 Kode Venkatadri Chawdhary Campus, L V Prasad Eye Institute, Vijayawada, India; 3 L V Prasad Image Laboratory and Analysis Centre, L V Prasad Eye Institute, Hyderabad, India; 4 Kallam Anji Reddy Campus, L V Prasad Eye Institute, Hyderabad, India; 5 Srimati Kanuri Santhamma Center for Vitreo Retinal Diseases, L V Prasad Eye Institute, Hyderabad, India

**Keywords:** acute, resolving and resolved posterior scleritis, OCT biomarkers, optical coherence tomography (OCT), posterior scleritis, retinal imaging

## Abstract

**Purpose:**

The study aimed to analyze the optical coherence tomography (OCT) characteristics in different stages of posterior scleritis (PS) and explore possible correlations between OCT features and the different stages of PS.

**Methods:**

This is a retrospective study on clinically diagnosed cases of PS with transparent media and good OCT images at an academic tertiary eye care center in India between April 2014 and March 2019. Stage-specific prevalence, distribution, and associations of 25 predefined OCT biomarkers across the acute, resolving, and resolved stages of PS were analyzed.

**Results:**

A total of 104 OCT plates from 31 patients were analyzed; 43, 28, and 33 were associated with active, resolving, and resolved stages of PS, respectively. Acute PS demonstrated choroidal thickening (100%), retinal pigment epithelium (RPE)–choroidal bump (97.7%), internal limiting membrane (ILM) folds (90.7%), and neurosensory detachment (NSD) (86.0%); resolving PS showed hyperreflective dot (HRD) prevalence (inner retina 92.9%, subretinal space 92.9%, and outer retina 89.3%); while resolved PS was characterized by ellipsoid zone (EZ) reflectivity (54.5%), variable external limiting membrane (ELM) reflectivity (51.5%), and epiretinal membrane (ERM) (39.4%). Median central foveal thickness (CFT) declined significantly from 737 µm in acute PS to 269 µm in resolving PS and 212 µm in resolved PS (*p* < 0.0001). Acute PS was strongly associated with RPE–choroidal bump [odds ratio (OR) 527.7, 95% confidence interval (CI) 54.8–5,079.4], ILM folds (OR 73.1, 95% CI 19.5–273.9), RPE thickening (OR 29.0, 95% CI 9.1–92.3), NSD (OR 14.8, 95% CI 5.0–44.0), cystoid macular edema (OR 8.3, 95% CI 3.4–20.5), and vitreous cells (OR 3.1, 95% CI 1.2–7.6). Resolving PS showed associations with HRD involving the inner retina (OR 8.39, 95% CI 1.95–36.2), outer retina (OR 7.10, 95% CI 1.94–25.9), subretinal space (OR 7.43, 95% CI 1.72–32.2), and choroid (OR 4.50, 95% CI 1.20–16.8). Resolved PS was associated with variable reflectivity of the EZ (OR 15.6, 95% CI 3.75–65.0) and ELM (OR 7.31, 95% CI 1.87–28.5), and posterior vitreous detachment (OR 2.80, 95% CI 1.29–6.11).

**Conclusion:**

OCT characteristics analyzed in this study can aid in identifying the stage of disease activity in PS.

## Introduction

Inflammation of the sclera involving adjacent episclera in the posterior segment of the eye is called posterior scleritis (PS) (1, 2). It can be potentially sight-threatening; thus, diagnosing the disease and initiating treatment early becomes crucial. Diagnosis of PS is based on clinical presentation, ultrasonography, and fundus fluorescein angiography (3–5). Other imaging modalities like computed tomography (CT) and magnetic resonance imaging (MRI) have also been proposed (6). However, because of the complex nature of the disease and the presence of features overlapping with other pathological conditions of the posterior pole, such as Vogt–Koyanagi–Harada (VKH) disease, central serous chorioretinopathy (CSCR), choroiditis, choroidal tumor, and optic neuritis, detection of multimodal imaging-based features specific to PS is of utmost importance (7, 8).

Optical coherence tomography (OCT) is a noninvasive way of analyzing the retina–choroidal complex, which can be used to understand the diagnosis, disease progression, and management. There are few anecdotal reports on OCT features of PS. Some of the OCT findings include subretinal fluid (SRF), punctate hyperreflective lesions in the choroid, choroidal thickening, retinal folds, macular and optic disc edema, and wave-like changes in retinal pigment epithelium (9–11). In the study, we aim to address various OCT findings that may be present in different stages of PS systematically. We also look for any possible correlation between the OCT characteristics and the different stages of PS.

## Methods

This was a retrospective chart review of prospectively collected data on PS between April 2014 and March 2019 at L V Prasad Eye Institute, Andhra Pradesh, India. Clinically diagnosed cases of PS with transparent media and good OCT pictures were included in this study. PS with other retinal pathology, poor image quality, recurrent PS, diagnosis of any other PS at any point, and intake of any anti-tubercular drug or any antibiotic for infectious pathology were excluded.

Data included age, gender, laterality, eye involvement, and imaging plate OCT characteristics. The OCT images (Triton, Topcon Swept Source OCT, and SD-OCT Zeiss 6000) were retrieved from the electronic medical record system and analyzed layer by layer by an expert image reader (SN). Good-quality images were included in the study.

Based on the clinical criteria and management, the OCT plates were segregated into three categories: acute PS, resolving PS, and resolved PS. Acute PS was a treatment-naïve case presented within 2 weeks of symptoms. Resolving PS was defined as diagnosed cases of PS at 2 weeks of starting oral corticosteroid treatment. Resolved PS was defined as when systemic therapy was stopped. Each patient underwent fundus photography, fundus fluorescein angiography, and OCT before treatment and whenever required.

Prior to image analysis, a standardized grading protocol was developed in which 25 OCT characteristics were pre-specified based on previously reported inflammatory retinal biomarkers, structural changes relevant to posterior segment inflammation, and expert consensus from vitreoretinal specialists (NS, RAK, RN, SN, and RN). These predefined parameters were selected before data extraction to ensure systematic and unbiased evaluation across all disease stages. All included OCT images were subsequently reviewed in a uniform layer-by-layer manner by an expert image reader (SN) according to this predetermined grading framework, with no *post hoc* modification of imaging variables during statistical analysis.

We studied 24 imaging characteristics in OCT; they were vitreous cells, posterior vitreous detachment (PVD), distorted foveal contour, central foveal thickness (CFT), internal limiting membrane (ILM) folds, epiretinal membrane (ERM), macular hole (MH), hyperreflective inner retina, hyperreflective outer retina, cystoid macular edema (CME), neurosensory detachment (NSD), hyperreflective dots (HRDs) in the inner retina, HRD in the outer retina, HRD in subretinal space, HRD in the choroid, variable reflectivity of the external limiting membrane (ELM), disrupted ELM, variable reflectivity of ellipsoid zone (EZ), disrupted EZ, RPE thickening, RPE thinning, pigment epithelial detachment (PED), RPE–choroidal bump, and choroidal thickening (CT).

### Stage-wise OCT biomarker prevalence analysis across PS

Stage-wise prevalence of predefined OCT biomarkers was calculated as the proportion of OCT images demonstrating each biomarker within acute, resolving, and resolved PS groups. Biomarker prevalence was expressed as percentages within each disease stage. Comprehensive stage-wise biomarker distribution was summarized using raw prevalence tables and visualized using heatmap analysis, where color intensity corresponded to biomarker prevalence percentage and comparative bar graphs to highlight structural transitions across stages of PS.

### Central foveal thickness analysis

CFT was measured quantitatively from OCT scans passing through the foveal center using device-generated calliper measurements from the ILM to the RPE. CFT values were recorded in micrometers (µm) and analyzed as a continuous variable across acute, resolving, and resolved PS groups. Stage-wise comparisons were performed using the Kruskal–Wallis test. Median values, interquartile ranges (IQRs), minimum values, and maximum values were calculated for each stage. Box-and-whisker plots were generated to visualize stage-wise CFT distribution, variability, and outlier patterns.

### Bivariate clustered generalized estimating equation analysis

To account for repeated OCT observations obtained from the same patient, stage-specific bivariate generalized estimating equation (GEE) logistic regression models with robust standard errors were constructed using patient identification number as the clustering variable. Separate binary comparisons were performed for acute PS versus non-acute stages, resolving PS versus non-resolving stages, and resolved PS versus non-resolved stages. Each predefined OCT biomarker was evaluated individually in separate bivariate models. Odds ratios (ORs), 95% confidence intervals (CIs), and *p*-values were calculated for each biomarker. An exchangeable correlation structure was assumed to account for within-patient correlation across repeated OCT observations.

### Statistical analysis

All statistical analyses were performed using Python (version 3.9). Categorical OCT biomarkers were summarized as frequencies and percentages across acute, resolving, and resolved PS stages. Continuous variables such as CFT were summarized using median and IQR. Because CFT demonstrated non-normal distribution, stage-wise comparisons were performed using the Kruskal–Wallis test. A two-tailed *p*-value <0.05 was considered statistically significant.

## Results

### Data set

**Figure 1:**
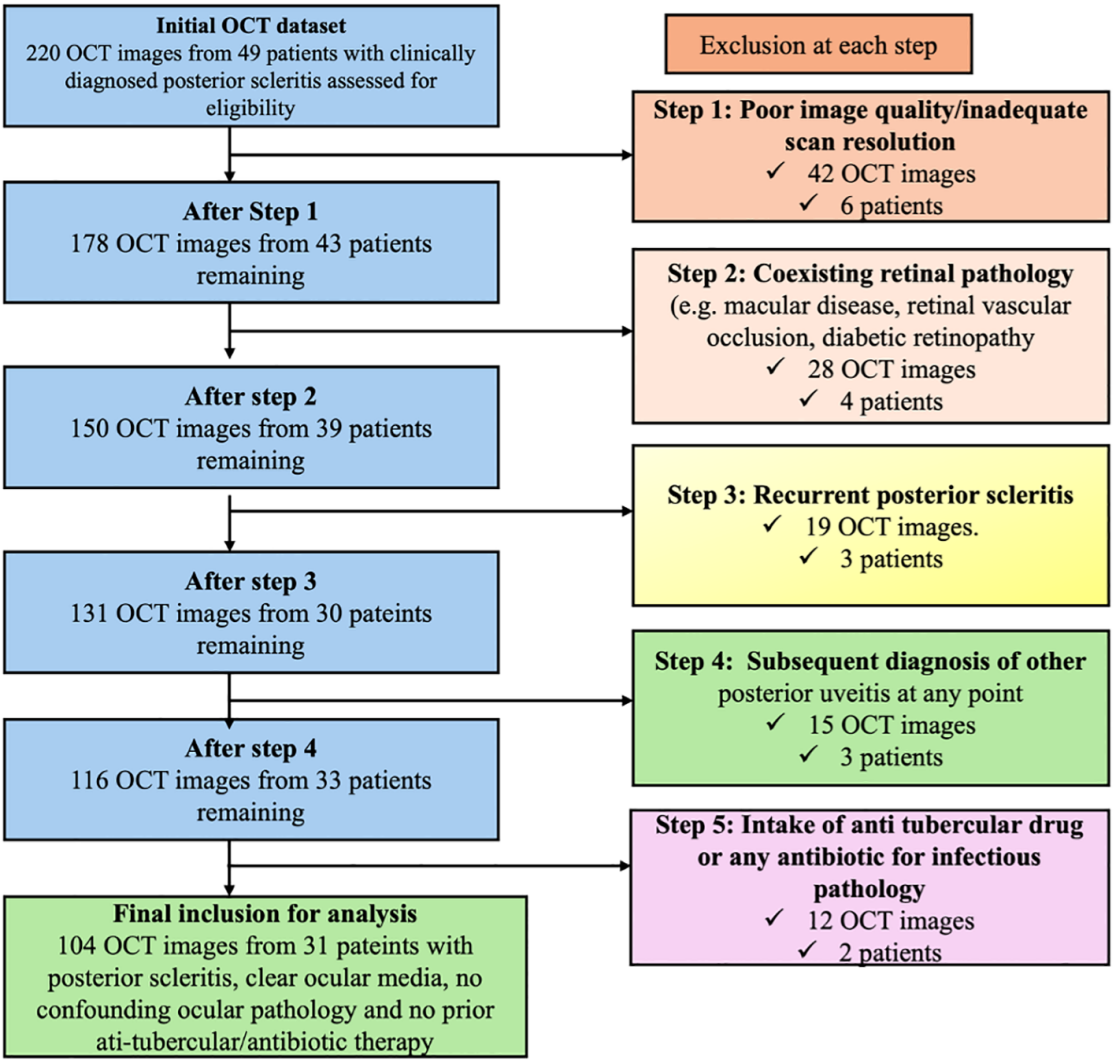
Flowchart of patient and optical coherence tomography (OCT) image selection. A total of 220 OCT images from 49 patients with clinically diagnosed posterior scleritis were assessed for eligibility. After stepwise exclusion based on predefined criteria, including poor image quality, coexisting retinal pathology, recurrent disease, alternative posterior uveitic diagnoses, and prior infectious therapy, 104 OCT images from 31 patients were included in the final analysis.

Among 220 OCT images obtained from 49 patients with clinically diagnosed PS, 116 images from 18 patients were excluded in a stepwise manner according to predefined criteria. Ultimately, 104 high-quality OCT images from 31 treatment-naïve patients with PS, clear ocular media, and no confounding retinal or infectious pathology were included for final analysis. A detailed patient selection flowchart summarizing inclusions and exclusions at each step is provided in **Figure 1**. **Table 1** shows the demographic characteristics.

**Table 1:** Baseline patient-level demographic, clinical, and OCT imaging characteristics of the posterior scleritis cohort.

Variable	Value
Total patients included	31
Total OCT images analyzed	104
Total unique patients contributing OCT images	32
Acute-stage OCT images, *n* (%)	43 (41.3)
Resolving-stage OCT images, *n* (%)	28 (26.9)
Resolved-stage OCT images, *n* (%)	33 (31.7)
Male sex, *n* (%)	11 (35.5)
Median age at presentation, years (range)	40 (13–60)
Bilateral posterior scleritis, *n* (%)	11 (35.5)
Right eye involvement only, *n* (%)	9 (29.0)
Median follow-up duration, days (range)	222 (14–1,287)
Systemic corticosteroid therapy, *n* (%)	31 (100)

The median presenting best-corrected visual acuity (BCVA) (Log MAR) was 0.60 (R: 0–1.69; SD: 0.53; Snellen equivalent 20/80) and median final BCVA (Log MAR) was 0.00 (R: 0–1.69; SD: 0.34; Snellen equivalent 20/20).

### Stage-wise OCT biomarker distribution across PS

**Figure 2:**
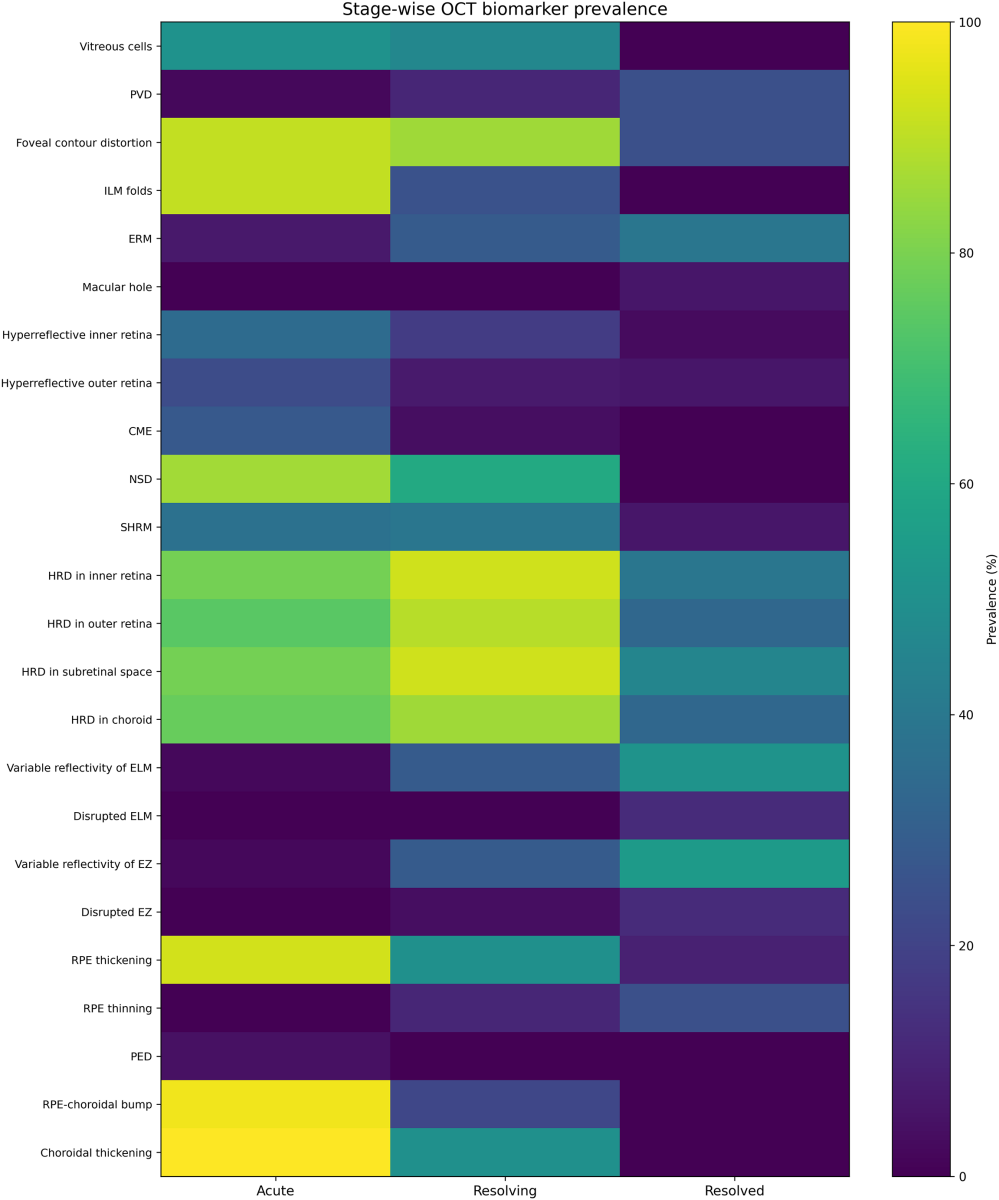
Stage-wise distribution of optical coherence tomography (OCT) biomarkers across acute, resolving, and resolved posterior scleritis (PS). Heatmap demonstrating prevalence (%) of all predefined OCT biomarkers across disease stages. Acute PS was characterized predominantly by inflammatory choroidal and retinal biomarkers including choroidal thickening, RPE–choroidal bump, ILM folds, neurosensory detachment, and RPE thickening. Resolving PS demonstrated peak prevalence of hyperreflective dots (HRDs) across retinal and choroidal layers, reflecting transitional inflammatory remodeling. Resolved PS was characterized by chronic structural sequelae including variable reflectivity and disruption of the external limiting membrane (ELM) and ellipsoid zone (EZ), epiretinal membrane (ERM), retinal pigment epithelium thinning, and posterior vitreous detachment (PVD).

**Figure 3:**
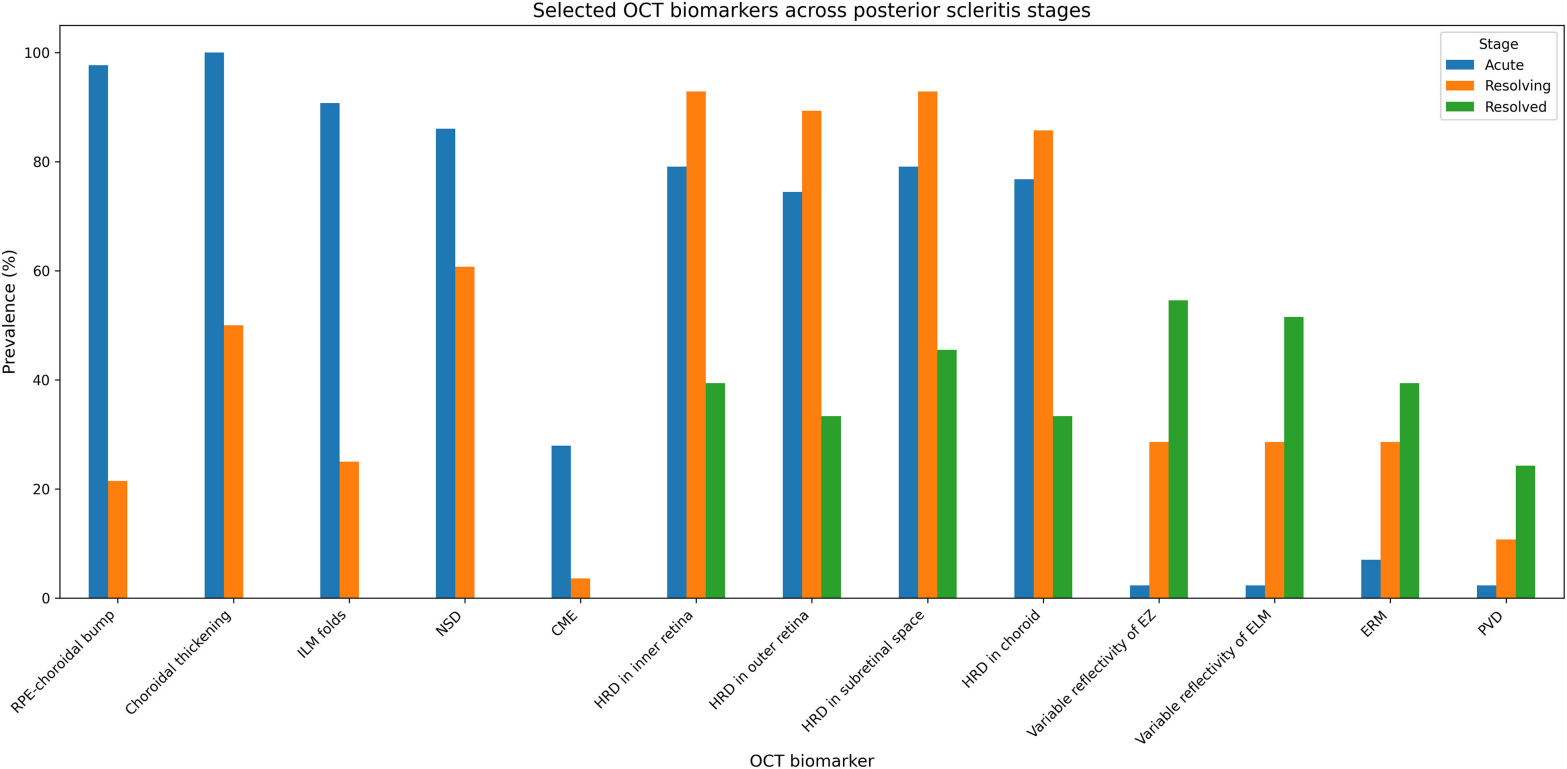
Dynamic changes in selected optical coherence tomography (OCT) biomarkers across acute, resolving, and resolved stages of posterior scleritis (PS). Bar graph of selected clinically relevant OCT biomarkers highlighting major stage-specific structural transitions. Acute PS demonstrated severe inflammatory edema, resolving PS demonstrated persistent hyperreflective dot (HRD) redistribution, and resolved PS demonstrated chronic outer retinal and vitreoretinal interface remodeling. These complementary visualizations illustrate progressive OCT phenotype evolution in PS.

Stage-wise OCT biomarker analysis demonstrated distinct structural phenotypes across acute, resolving, and resolved posterior scleritis, reflecting progressive transition from active inflammation to chronic retinal remodeling (**Table 2**; **Figures 2**, **3**).

**Table 2:** Stage-wise prevalence of OCT biomarkers across posterior scleritis stages.

Biomarker	Acute *n*/*N* (%)	Resolving *n*/*N* (%)	Resolved *n*/*N* (%)
Vitreous cells	22/43 (51.2)	13/28 (46.4)	0/33 (0.0)
Posterior vitreous detachment (PVD)	1/43 (2.3)	3/28 (10.7)	8/33 (24.2)
Distorted foveal contour	39/43 (90.7)	24/28 (85.7)	8/33 (24.2)
ILM folds	39/43 (90.7)	7/28 (25.0)	0/33 (0.0)
Epiretinal membrane (ERM)	3/43 (7.0)	8/28 (28.6)	13/33 (39.4)
Macular hole (MH)	0/43 (0.0)	0/28 (0.0)	2/33 (6.1)
Hyperreflective inner retina	15/43 (34.9)	5/28 (17.9)	1/33 (3.0)
Hyperreflective outer retina	10/43 (23.3)	2/28 (7.1)	2/33 (6.1)
Cystoid macular edema (CME)	12/43 (27.9)	1/28 (3.6)	0/33 (0.0)
Neurosensory detachment (NSD)	37/43 (86.0)	17/28 (60.7)	0/33 (0.0)
Subretinal hyperreflective material (SHRM)	16/43 (37.2)	11/28 (39.3)	2/33 (6.1)
Hyperreflective dots in inner retina	34/43 (79.1)	26/28 (92.9)	13/33 (39.4)
Hyperreflective dots in outer retina	32/43 (74.4)	25/28 (89.3)	11/33 (33.3)
Hyperreflective dots in subretinal space	34/43 (79.1)	26/28 (92.9)	15/33 (45.5)
Hyperreflective dots in choroid	33/43 (76.7)	24/28 (85.7)	11/33 (33.3)
Variable reflectivity of ELM	1/43 (2.3)	8/28 (28.6)	17/33 (51.5)
Disrupted ELM	0/43 (0.0)	0/28 (0.0)	4/33 (12.1)
Variable reflectivity of EZ	1/43 (2.3)	8/28 (28.6)	18/33 (54.5)
Disrupted EZ	0/43 (0.0)	1/28 (3.6)	4/33 (12.1)
RPE thickening	40/43 (93.0)	14/28 (50.0)	3/33 (9.1)
RPE thinning	0/43 (0.0)	3/28 (10.7)	8/33 (24.2)
Pigment epithelial detachment (PED)	2/43 (4.7)	0/28 (0.0)	0/33 (0.0)
RPE–choroidal bump	42/43 (97.7)	6/28 (21.4)	0/33 (0.0)
Choroidal thickening	43/43 (100.0)	14/28 (50.0)	0/33 (0.0)

ILM, internal limiting membrane; ERM, epiretinal membrane; CME, cystoid macular edema; NSD, neurosensory detachment; SHRM, subretinal hyperreflective material; ELM, external limiting membrane; EZ, ellipsoid zone; RPE, retinal pigment epithelium; PED, pigment epithelial detachment; PVD, posterior vitreous detachment.

**Figure 4:**
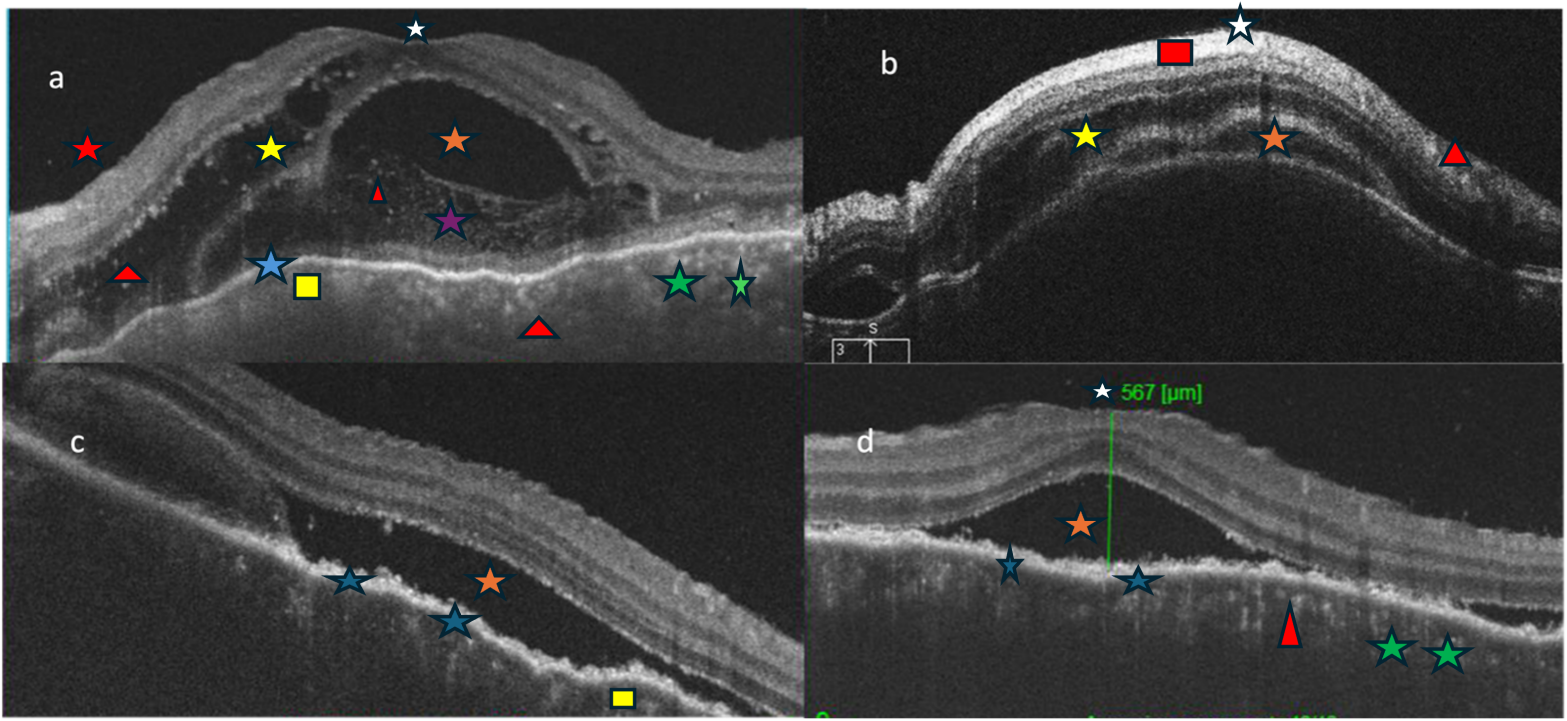
Optical coherence tomography (OCT) in acute posterior scleritis (PS). OCT picture showing occasional vitreous cells (red star, a); distorted foveal contour (white star, a, b, d); cystoid changes in the retina (yellow star, **(a, b)**; neurosensory detachment (orange star, **(a–d)**; hyperreflective material in subretinal space (purple star, a); choroidal bump (yellow boxes, **(a, c)**; thickened RPE (blue stars, **(a, c, d)**; thickened choroid (green stars, **(a, d)**. **(a)**; and hyperreflective inner retina (red box, **(b)**. The hyperreflective dots (HRDs) in the inner retina, outer retina, subretinal space, or in the choroid (red triangles, **(a–c)**.

Acute PS was predominantly characterized by inflammatory and choroidal biomarkers, including choroidal thickening, RPE–choroidal bump, RPE thickening, ILM folds, distorted foveal contour, and NSD. HRDs involving the inner retina, outer retina, subretinal space, and choroid were also commonly observed during acute disease (**Figure 4**).

**Figure 5:**
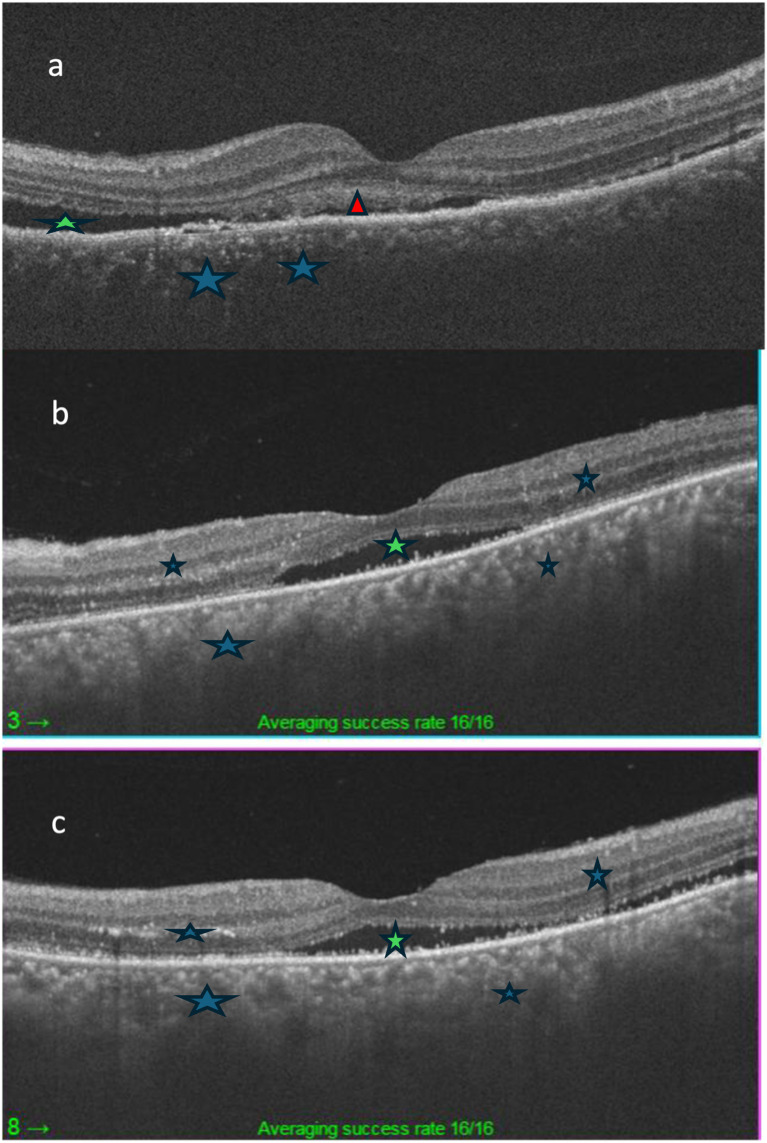
Optical coherence tomography (OCT) in resolving posterior scleritis (PS). Resolving stage of PS showing shallow neurosensory detachment (green star, **(a–c)**; minimal to absent cystoid macular edema **(a–c)**; subretinal hyperreflective material (red triangle, **(a)**; and hyperreflective dots predominantly in the outer plexiform layer (blue stars, **(c)**.

During the resolving stage, acute inflammatory biomarkers declined substantially, while HRD biomarkers demonstrated peak prevalence, suggesting ongoing inflammatory debris redistribution and transitional retinal remodeling during corticosteroid-mediated recovery (**Figure 5**).

**Figure 6:**
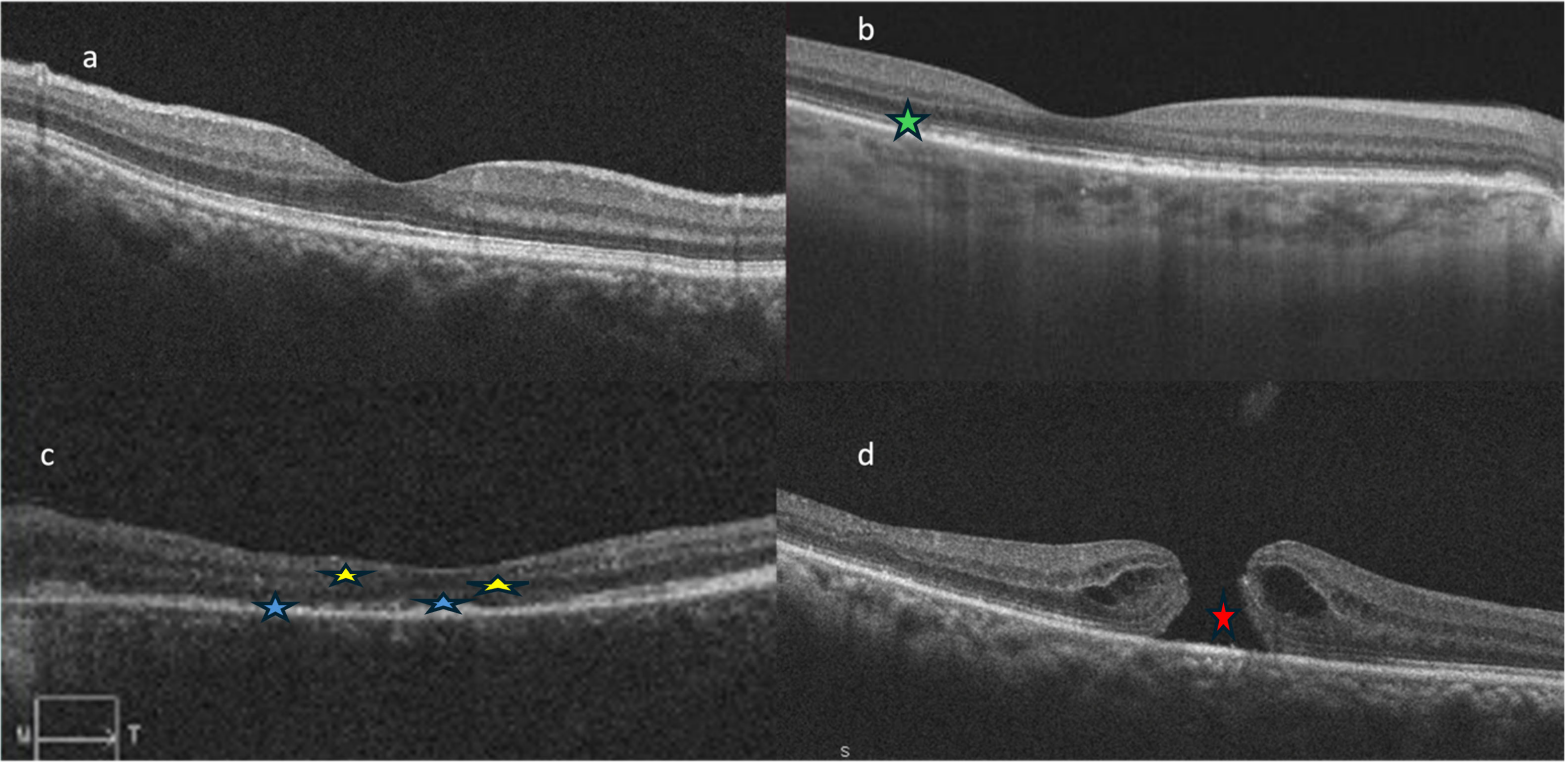
Optical coherence tomography (OCT) in resolved posterior scleritis (PS). Resolved PS with complete restoration of anatomy, intact inner and outer retina **(a)**, with altered reflectivity of ellipsoid zone (green star, **(b)**, with thinning of the inner retina (yellow stars, **(c)** and disrupted external limiting membrane and ellipsoid zone (blue stars, **(c)**, and with full-thickness macular hole (red star, **(d)**.

Resolved PS demonstrated near-complete disappearance of acute inflammatory biomarkers. Chronic structural remodeling changes became predominant, including variable EZ and ELM reflectivity, ERM formation, PVD, and RPE thinning, indicating persistent outer retinal and vitreoretinal interface abnormalities (**Figure 6**).

### Central foveal thickness analysis

**Figure 7:**
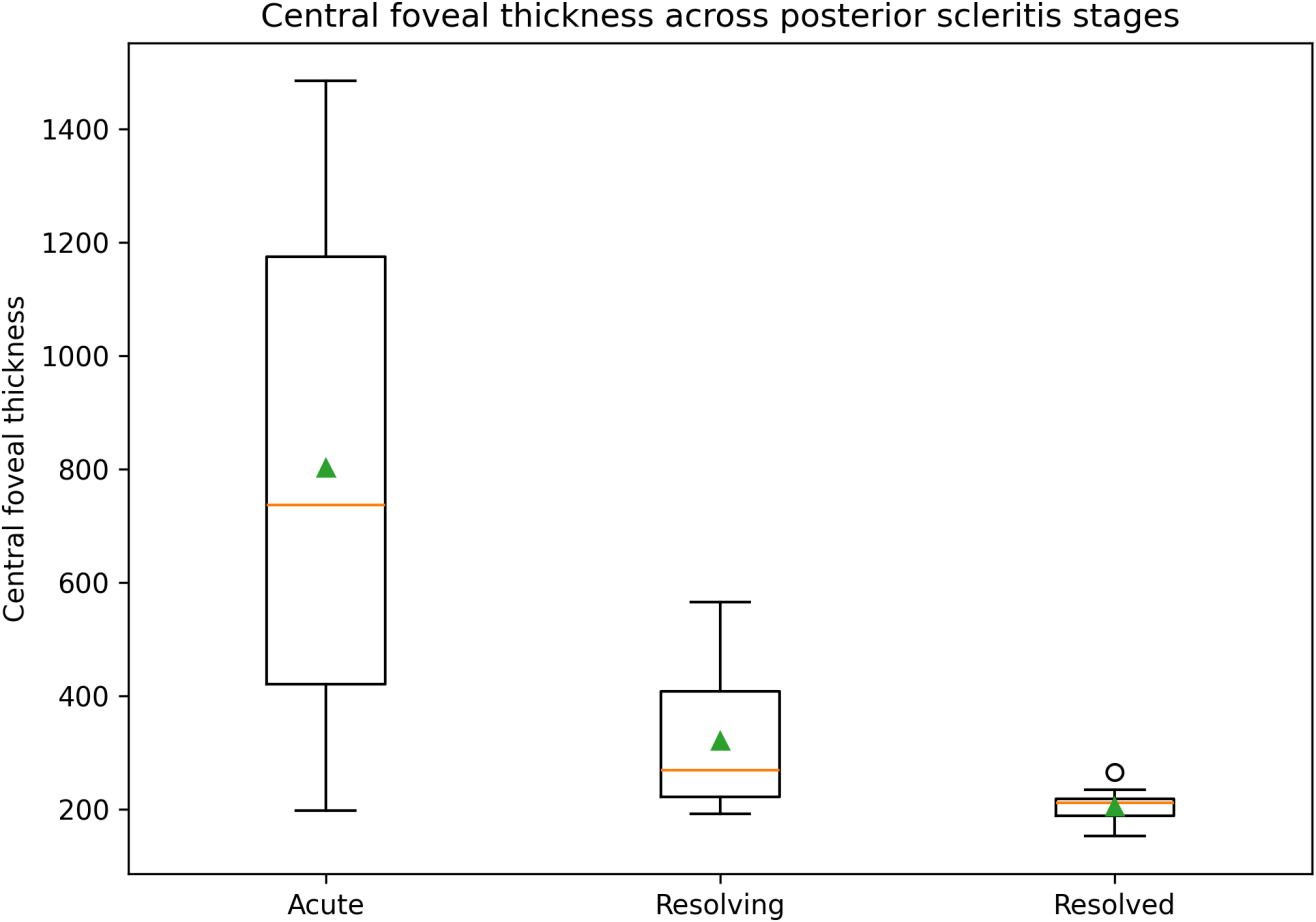
Box-and-whisker plot demonstrating central foveal thickness (CFT) across acute, resolving, and resolved posterior scleritis (PS) stages. Acute PS demonstrated markedly elevated CFT, reflecting severe inflammatory retinal edema and structural distortion. Resolving PS showed substantial reduction in CFT, indicating treatment response and regression of exudative changes. Resolved PS demonstrated near normalization of CFT with minimal variability, consistent with anatomical recovery. Horizontal lines within boxes represent medians, boxes represent interquartile ranges, whiskers indicate overall distribution, and green triangles denote mean values. This image highlights progressive normalization of macular architecture with PS resolution.

CFT demonstrated a marked stage-dependent decline across PS stages (**Figure 7**). Acute PS showed the greatest retinal thickening, with a median CFT of 737 µm (IQR: 420.5–1,174.5 µm), compared with 269 µm (IQR: 222.3–408.3 µm) in resolving disease and 212 µm (IQR: 189–218 µm) in resolved disease. Acute-stage OCT images also demonstrated the widest variability in retinal thickness (range: 198–1,485 µm), reflecting heterogeneous inflammatory edema severity. In contrast, resolved PS demonstrated substantially lower CFT values and a narrower distribution range (153–265 µm), indicating resolution of inflammatory retinal edema. Overall stage-wise differences in CFT were highly significant (Kruskal–Wallis statistic = 44.19, *p* < 0.001).

### Bivariate clustered GEE analysis

Bivariate GEE analysis demonstrated distinct stage-specific OCT biomarker associations across acute, resolving, and resolved posterior scleritis.

Acute PS was strongly associated with inflammatory and choroidal biomarkers, including RPE–choroidal bump (OR 527.7, 95% CI 54.8–5079.4; *p* < 0.001), ILM folds (OR 73.1, 95% CI 19.5–273.9; *p* < 0.001), RPE thickening (OR 29.0, 95% CI 9.1–92.3; *p* < 0.001), NSD (OR 14.8, 95% CI 5.0–44.0; *p* < 0.001), CME (OR 8.3, 95% CI 3.4–20.5; *p* < 0.001), and vitreous cells (OR 3.1, 95% CI 1.2–7.6; *p* = 0.015). In contrast, chronic remodeling biomarkers such as ERM, variable reflectivity of the ELM, and variable reflectivity of the EZ demonstrated significantly lower odds during acute disease.

Resolving PS demonstrated strongest associations with HRD biomarkers. HRDs in the inner retina (OR 8.39, 95% CI 1.95–36.2; *p* = 0.004), outer retina (OR 7.10, 95% CI 1.94–25.9; *p* = 0.003), subretinal space (OR 7.43, 95% CI 1.72–32.2; *p* = 0.007), and choroid (OR 4.50, 95% CI 1.20–16.8; *p* = 0.026) were significantly associated with resolving-stage disease. Distorted foveal contour also showed increased odds during the resolving phase (OR 1.95, 95% CI 1.13–3.35; *p* = 0.016). Conversely, ILM folds (OR 0.31, 95% CI 0.14–0.71; *p* = 0.006), hyperreflective outer retina (OR 0.35, 95% CI 0.13–0.90; *p* = 0.030), and RPE–choroidal bump (OR 0.22, 95% CI 0.06–0.73; *p* = 0.013) demonstrated lower odds during the resolving stage.

Resolved PS was predominantly associated with chronic outer retinal and vitreoretinal interface remodeling biomarkers. Variable reflectivity of the EZ demonstrated the strongest association with resolved-stage disease (OR 15.6, 95% CI 3.75–65.0; *p* < 0.001), followed by variable reflectivity of the ELM (OR 7.31, 95% CI 1.87–28.5; *p* = 0.004) and PVD (OR 2.80, 95% CI 1.29–6.11; *p* = 0.010). In contrast, acute inflammatory biomarkers including RPE thickening (OR 0.037, 95% CI 0.013–0.111; *p* < 0.001), choroidal thickening (*p* < 0.001), ILM folds (*p* < 0.001), NSD (*p* < 0.001), and distorted foveal contour (*p* < 0.001) demonstrated markedly reduced odds during resolved-stage disease. These findings indicate a progressive transition from inflammatory edema and choroidal swelling toward chronic outer retinal and vitreoretinal remodeling during disease resolution.

## Discussion

Posterior scleritis demonstrates a dynamic spectrum of retinal, choroidal, and vitreoretinal interface alterations on OCT that evolve with disease activity and treatment response. In this study, stage-wise OCT biomarker analysis demonstrated distinct structural phenotypes across acute, resolving, and resolved PS. Acute-stage disease was predominantly characterized by inflammatory and choroidal biomarkers, including choroidal thickening, RPE–choroidal bump, ILM folds, distorted foveal contour, NSD, and increased CFT, reflecting active inflammatory edema and choroidal congestion. During the resolving stage, acute inflammatory biomarkers declined substantially, whereas HRD in the inner retina, outer retina, subretinal space, and choroid demonstrated peak prevalence, suggesting ongoing inflammatory debris redistribution and transitional retinal remodeling during corticosteroid-mediated recovery. In contrast, resolved PS demonstrated near-complete disappearance of acute inflammatory biomarkers, with chronic structural remodeling features such as variable EZ and ELM reflectivity, ERM formation, PVD, and RPE thinning becoming predominant. Bivariate clustered GEE analysis further demonstrated significant stage-specific biomarker associations, supporting the concept of a progressive transition from acute inflammatory edema and choroidal swelling toward chronic outer retinal and vitreoretinal remodeling during disease resolution.

The studies reported earlier have primarily focused on clinical features and ultrasonographic criteria of PS (2–5, 7, 12, 13). NSD was found to be associated with 62% and 50% of PS in studies by Ando and Agrawal et al., respectively (4, 14). In our series in the acute stage, we found NSD in 86.02%. In our case, the increased prevalence of NSD could be related to OCT-based diagnosis of NSD, whereas, in previous studies, NSD was diagnosed based on USG. OCT features like increased choroidal thickness, hyperreflective RPE, CME, NSD, and HRDs have been reported in some case reports and anecdotal short case series (4, 7–12, 14).

Histopathology of the enucleated eye of PS had shown the involvement of sclera, choroid, and retina (3). Sclera and choroid showed inflammation, infiltration of acute and chronic inflammatory cells, and vasculitis (3). Retinal pigment epithelium disruption was also a feature in histopathology; we found disruption of ELM and EZ in resolving and resolved stage of the disease (7). This is the first case series of posterior scleritis describing different characteristics of OCT features in different stages of the disease in a systematic manner very similar to histopathological findings.

The major limitation of the study is the lack of image-based software and automated segmentation to detect OCT biomarkers. Secondly, the OCT plates we have included were line scans, not volume scans. The study lacks comparative data on VKH disease/syndrome and CSCR, which are strong mimics of PS. Other limitations included a small sample size, non-blinding of the image reader to the disease state, and the exclusion of infectious causes of scleritis.

In conclusion, this study provides a comprehensive stage-wise OCT characterization of PS and highlights the dynamic structural evolution of the disease during treatment and resolution. The identification of distinct inflammatory, HRD, and chronic remodeling biomarkers across disease stages may improve understanding of PS pathophysiology and assist in objective imaging-based disease assessment. The use of clustered GEE analysis allowed the evaluation of OCT biomarkers while accounting for repeated measurements from individual patients, thereby strengthening the reliability of stage-specific associations. These findings may contribute toward the development of standardized OCT-based biomarkers for disease monitoring and therapeutic response assessment in PS.

## Data Availability

The datasets generated and/or analyzed during the current study are available from the corresponding author on reasonable request, subject to institutional policies and ethical approval.
